# Cabozantinib in advanced renal cell carcinoma: A phase II, open‐label, single‐arm study of Japanese patients

**DOI:** 10.1111/iju.14329

**Published:** 2020-08-12

**Authors:** Yoshihiko Tomita, Katsunori Tatsugami, Noboru Nakaigawa, Takahiro Osawa, Mototsugu Oya, Hiroomi Kanayama, Chihiro Nakayama Kondoh, Naoto Sassa, Kazuo Nishimura, Masahiro Nozawa, Naoya Masumori, Yasuhide Miyoshi, Shingo Kuroda, Shingo Tanaka, Akiko Kimura, Satoshi Tamada

**Affiliations:** ^1^ Department of Urology, Molecular Oncology Niigata University Graduate School of Medical and Dental Sciences Niigata Japan; ^2^ Department of Urology Graduate School of Medical Sciences Kyushu University Fukuoka Japan; ^3^ Department of Urology Yokohama City University Graduate School of Medicine Yokohama Japan; ^4^ Department of Renal and Genitourinary Surgery Hokkaido University Sapporo Japan; ^5^ Department of Urology Keio University School of Medicine Tokyo Japan; ^6^ Department of Urology Graduate School of Biomedical Sciences Tokushima University Tokushima Japan; ^7^ Department of Medical Oncology Toranomon Hospital Tokyo Japan; ^8^ Department of Urology Nagoya University Graduate School of Medicine Nagoya Japan; ^9^ Department of Urology Osaka International Cancer Institute Osaka Japan; ^10^ Department of Urology Kindai University Faculty of Medicine Osaka‐sayama Japan; ^11^ Department of Urology Sapporo Medical University School of Medicine Sapporo Japan; ^12^ Department of Urology and Renal Transplantation Yokohama City University Medical Center Yokohama Japan; ^13^ Biostatistics Japan Development Center Takeda Pharmaceutical Company Limited Osaka Japan; ^14^ PRA Health Sciences Company Limited Osaka Japan; ^15^ Oncology Clinical Science Oncology Therapeutic Area Unit for Japan and Asia Takeda Pharmaceutical Company Limited Tokyo Japan; ^16^ Department of Urology Osaka City University Graduate School of Medicine Osaka Japan

**Keywords:** cabozantinib, Japan, receptor tyrosine kinase, renal cell carcinoma, tyrosine kinase inhibitor

## Abstract

**Objectives:**

To evaluate the efficacy and safety of cabozantinib, through a bridging study to METEOR, in Japanese patients with advanced renal cell carcinoma who had progressed after prior tyrosine kinase inhibitor therapy.

**Methods:**

This phase II, open‐label, single‐arm study (ClinicalTrials.gov registration number: NCT03339219) included adult Japanese patients with advanced renal cell carcinoma and measurable disease who had received one or more tyrosine kinase inhibitors. Patients received cabozantinib 60 mg orally once daily while there was clinical benefit, or until unacceptable toxicity or disease progression. The primary end‐point was objective response rate per Response Evaluation Criteria in Solid Tumors Version 1.1. Secondary end‐points included clinical benefit rate (complete or partial response, or ≥8‐week stable disease), progression‐free survival, overall survival and safety.

**Results:**

Of the 35 patients enrolled, 68.6%, 22.9% and 8.6% had previously received one, two and three prior tyrosine kinase inhibitors, respectively. The median duration of cabozantinib exposure was 27.0 weeks (range 5.1–43.0 weeks). The objective response rate was 20.0% (90% confidence interval 9.8–34.3%), and the clinical benefit rate was 85.7% (95% confidence interval 69.7–95.2%). The 6‐month estimated progression‐free survival was 72.3% (95% confidence interval 53.3–84.6%); the median progression‐free survival and overall survival were not reached. All patients reported adverse events, which were manageable by supportive treatment or dose modification; two patients (5.7%) discontinued therapy due to adverse events.

**Conclusions:**

The results showed that findings from METEOR can be extrapolated, and that cabozantinib 60 mg/day is a viable treatment option in Japanese patients with advanced renal cell carcinoma who had progressed after prior tyrosine kinase inhibitor therapy.

**Abbreviations & Acronyms**
AE: adverse event
CBR: clinical benefit rate
CI: confidence interval
CR: complete response
CT: computed tomography
HR: hazard ratio
IRC: independent radiology committee
MRI: magnetic resonance imaging
ORR: objective response rate
OS: overall survival
PD‐1: programmed cell
PD‐L1/L2: programmed cell death ligand 1/2
PFS: progression‐free survival
PK: pharmacokinetics
PR: partial response
RCC: renal cell carcinoma
RECIST 1.1: Response Evaluation Criteria in Solid Tumors Version 1.1
SD: stable disease
TEAE: treatment‐emergent adverse event
TKI: tyrosine kinase inhibitor
VEGF: vascular endothelial growth factor
VEGFR: vascular endothelial growth factor receptor

## Introduction

In a 2018 report, there were an estimated 31 700 cancer cases in the kidney and other urinary organs (excluding bladder) in Japan (3% of all cancers), accounting for 4622 deaths (1.2% of all cancer deaths).^1^ The 5‐year relative survival rate for this group was 69.1%.^1^

Approximately 90%^2^ of kidney cancers are RCC, and 65–75%^3^, ^4^ of these have a clear cell histology. Clear cell RCC commonly shows mutations in the tumor suppressor Von Hippel–Lindau gene, triggering a decrease in the degradation of hypoxia‐inducible factor, and an increase in VEGF transcription and tumor angiogenesis.^5^ VEGFR2 is a key mediator of VEGF signaling in tumor angiogenesis.^5^, ^6^ Resistance to VEGF‐targeted therapies might arise from the upregulation of alternative pro‐angiogenic and pro‐invasive signaling pathways, including the MET and AXL pathways.^7^ Therefore, effective therapies after VEGF‐targeted TKI therapies are needed. Recent data suggest that nivolumab–ipilimumab, pembrolizumab–axitinib or avelumab–axitinib offered clinical benefit over sunitinib and should be considered as standard therapy in candidates for immunotherapy.^8^, ^9^, ^10^ However, the optimal therapy after immune checkpoint inhibitor‐containing regimens is unknown.

Cabozantinib is a multiple receptor tyrosine kinase inhibitor targeting MET (c‐MET), VEGFR2, RET, AXL, KIT and TIE‐2, which are implicated in tumor growth, metastasis and angiogenesis.^6^ In the phase III, randomized, open‐label METEOR study (NCT01865747), cabozantinib was associated with a significant PFS benefit (7.4 *vs* 3.8 months; HR 0.51, 95% CI 0.41–0.62, *P* < 0.0001) and a greater ORR (17% *vs* 3%; *P* < 0.0001) versus everolimus in patients with metastatic RCC who had progressed after VEGFR‐TKI.^11^, ^12^ Significant OS benefit with cabozantinib over everolimus was also observed (21.4 *vs* 16.5 months, *P* = 0.00026).^12^ In the phase II CABOSUN trial (NCT01835158), cabozantinib also showed PFS benefit (8.2 *vs* 5.6 months) and reduced the rate of disease progression or death by 34% (HR 0.66, 95% CI 0.46–0.95, *P = *0.012) over first‐line sunitinib.^13^ The safety and tolerability of cabozantinib was investigated in a Japanese phase I study (NCT01553656) of patients with advanced solid tumors (predominantly non‐small cell lung cancer); that study reported a comparable safety profile to that seen in Western patients.^14^

The current study, designed to bridge the results from METEOR, which does not include patients in Japan, assessed the efficacy and safety of cabozantinib in Japanese patients with advanced RCC who had progressed after treatment with a prior VEGFR‐TKI.

## Methods

### Study design

This phase II, open‐label, single‐arm study (ClinicalTrials.gov registration number: NCT03339219), carried out at 19 sites, evaluated the efficacy and safety of cabozantinib in Japanese patients with advanced RCC who had progressed after prior VEGFR‐TKI therapy. The primary end‐point was ORR assessed by IRC. Secondary end‐points included CBR, PFS, OS and safety. An exploratory end‐point was plasma PK.

### Patients

Eligible patients were Japanese, aged ≥20 years with a documented histologic or cytologic diagnosis of RCC with a clear cell component and measurable disease per RECIST 1.1, as determined by the investigator. Patients must have received one or more VEGFR‐TKIs (e.g. sorafenib, sunitinib, axitinib, pazopanib), with the most recent dose received within 6 months before the first study drug administration, and had radiographically documented progression during treatment or received treatment for ≥4 weeks and radiographically progressed within 6 months after the last dose. Patients who had received an immune checkpoint inhibitor were also eligible. Other inclusion criteria were as follows: recovery from toxicities related to any prior treatments to baseline or grade ≤1 Common Terminology Criteria for Adverse Events Version 4.03 (unless AEs were clinically non‐significant and/or stable on supportive therapy); Karnofsky Performance Status score of ≥70%; and adequate organ and marrow function at screening.

Key exclusion criteria were as follows: prior treatment with everolimus, or other specific or selective target of rapamycin complex 1/phosphoinositide 3‐kinase/AKT inhibitor (e.g. temsirolimus), or cabozantinib; treatment with any small‐molecule kinase inhibitor, including investigational kinase inhibitors within 14 days; or treatment with any anticancer antibody within 28 days before the first dose of the study drug. Those with uncontrolled, significant intercurrent or recent illness, active infection, recent major surgery, concomitant anticoagulation (with oral anticoagulants or platelet inhibitor), or who were pregnant, breastfeeding or not practicing contraception were ineligible.

### Study drug

Eligible patients received cabozantinib 60 mg orally once‐daily in a fasted state. Cabozantinib dose modifications (reductions or interruptions) were allowed for AEs, abnormalities in laboratory assessments or other toxicity. Patients received the study drug for as long as they experienced investigator‐determined clinical benefit, or until there was unacceptable toxicity or disease progression requiring subsequent anticancer therapy. Investigators could elect to the continue study drug after radiographic RCC progression per RECIST 1.1, provided the participant was still deriving clinical benefit. However, treatment was discontinued after any second determination of disease progression. Post‐treatment follow up was carried out up to 30 days after the last dose of the study drug or until the start of subsequent anticancer therapy, whichever occurred first.

### Efficacy and safety assessments

Response and disease progression were determined by RECIST 1.1. Tumor assessments for chest/abdomen/pelvis were carried out by CT or MRI at screening and every 8 weeks (±7 days) after the first dose of the study drug. Brain MRI or CT was carried out at the same timing in those with brain metastases. Technetium bone scans were carried out at screening, and then every 16 weeks (±7 days) after the first study drug dose in those with bone metastases.

A central IRC reviewed and classified all images for assessing efficacy. ORR was defined as the proportion of patients whose best overall response was CR or PR per RECIST 1.1, which was confirmed by a subsequent evaluation carried out ≥28 days later. Only results of tumor assessment, carried out on or before the earlier of the date of the PFS event or date of censoring for PFS by IRC, were used to determine the best overall response. CBR was defined as the proportion of patients whose best overall response was CR, PR or SD per RECIST 1.1. Any CR and PR required confirmation by a subsequent evaluation carried out ≥28 days later, and an assessment of SD was required to be ≥8 weeks (≥51 days) after the first day of study drug administration. PFS was defined as the time from the first day of the study drug administration to the earliest occurrence of progressive disease per RECIST 1.1 or death due to any cause. OS was defined as the time from the first day of study drug administration to death due to any cause.

TEAEs were coded using the Medical Dictionary for Regulatory Activities (version 21.0). Safety end‐points included TEAEs, grade ≥3 or serious TEAEs, permanent discontinuation or dose modification due to TEAEs, or clinically significant abnormal laboratory values or vital signs.

### PK assessment

On the first day of drug administration, PK blood samples were taken before and 3 h after dosing. Scheduled on‐treatment PK samples were obtained at weeks 3, 5 and 9 irrespective of whether the study drug was administered on that day. For each on‐treatment visit, the PK sample was collected approximately ≥8 h after the previous dose of study drug. If the study drug was administered on that day, then it was collected before administration. Plasma concentration of cabozantinib was analyzed using a validated bioanalytical method.

### Ethics and informed consent

The study protocol and associated documentation were reviewed by institutional review boards at each site. This study was carried out in compliance with the Declaration of Helsinki, the International Council for Harmonization Harmonized Guideline for Good Clinical Practice and all applicable local regulations. All patients provided written informed consent before enrollment.

### Statistical analysis

The planned enrolment of 35 patients allowed a 10% dropout rate to result in 32 evaluable patients providing 80% power in a binomial test to detect an ORR of ≥17% when testing a null hypothesis of ORR ≤3% at a one‐sided significance level of 5%.^12^ Extrapolating results from the METEOR study to Japanese patients was considered appropriate if the current study met this efficacy criterion and no unexpected major safety concern was reported.

Efficacy analyses were carried out using the full analysis set, comprising all patients receiving one or more doses of study drug. For ORR, point estimates and two‐sided 90% exact CI were provided. Time‐to‐tumor response was defined as the time from the first day of study drug administration to the first confirmed CR or PR, and was descriptively summarized. For CBR per RECIST 1.1, point estimates and two‐sided 95% exact CI were calculated. PFS and OS were estimated using the Kaplan–Meier method. Safety analyses were carried out for all patients who received one or more doses of study drug. Safety and PK results were descriptively summarized. All statistical analyses were carried out using SAS version 9.4 (SAS Institute, Cary, NC, USA).

## Results

### Patients

In total, 46 patients were screened and 35 received cabozantinib (10 failed screening, one did not enroll following physician decision). All patients had stage IV disease; 18 patients (51.4%) had three or more involved organs (Table 1). The most common sites were the lungs (60.0%), lymph nodes (31.4%), liver (25.7%), and bones (22.9%).

**Table 1:** Participant baseline characteristics (full analysis set/safety analysis set)

Characteristic	*n* = 35
Median age, years (range)	63.0 (42–84)
Sex, *n* (%)	
Male	24 (68.6)
Female	11 (31.4)
Memorial Sloan Kettering Cancer Center risk factor, *n* (%)	
Favorable	11 (31.4)
Intermediate	19 (54.3)
Poor	5 (14.3)
IMDC criteria, *n* (%)	
Favorable	6 (17.1)
Intermediate	22 (62.9)
Poor	7 (20.0)
Karnofsky performance status, *n* (%)	
70%	1 (2.9)
80%	5 (14.3)
90%	5 (14.3)
100%	24 (68.6)
Eastern Cooperative Oncology Group performance status, *n* (%)	
0	26 (74.3)
1	9 (25.7)
2	0
Median time from diagnosis to enrollment, years (range)	3.6 (0–17)
No. involved organs per IRC, *n* (%)	
1	6 (17.1)
2	11 (31.4)
≥3	18 (51.4)
Extent of baseline disease, *n* (%)	
Bone (CT or MRI)	8 (22.9)
Lung	21 (60.0)
Liver	9 (25.7)
Lung or liver	25 (71.4)
Lung or liver, and bone (CT or MRI)	4 (11.4)
Brain	0
Lymph node	11 (31.4)
Kidney	9 (25.7)
Other	15 (42.9)
Prior nephrectomy, *n* (%)	34 (97.1)
Prior radiation therapy, *n* (%)	9 (25.7)
Median number of prior systemic non‐radiation anticancer agents, *n* (range)	2.0 (1–8)
No. prior VEGFR‐TKI agents, *n* (%)	
1	24 (68.6)
2	8 (22.9)
≥3	3 (8.6)
Type of prior VEGFR‐TKI agents, *n* (%)	
Sunitinib	24 (68.6)
Axitinib†	18 (51.4)
Pazopanib	7 (20.0)
Sorafenib	0
Other VEGFR‐TKI agents	0
Prior therapy with anti‐PD‐1, anti‐PD‐L1/L2 agents	15 (42.9)
Nivolumab	11 (31.4)
Avelumab	3 (8.6)
Pembrolizumab	1 (2.9)

† Included the three patients who received prior avelumab, and one patient who received prior pembrolizumab.

A total of 24 patients (68.6%) had received one prior VEGFR‐TKI, and eight (22.9%) received two. The most common prior VEGFR‐TKIs were sunitinib (68.6%) and axitinib (51.4%). A total of 15 patients (42.9%) had received immuno‐oncology agents. Nivolumab (31.4%) was the most commonly used PD‐1‐targeting agent.

### Study drug administration

The median duration of cabozantinib exposure was 27.0 (range 5.1–43.0) weeks. The median daily dose was 26.0 mg (range 13.7–60.0 mg) with a median dose intensity of 43.4% (range 22.9–100.0%). At data cut‐off, 11 patients (31.4%) had discontinued study treatment. The most common reasons for discontinuation were progressive disease (5/11; 45.5%) and AE (2/11; 18.2%). Other discontinuations were attributed to death, clinical deterioration, withdrawal of informed consent and withdrawal by physician.

### Efficacy assessment

**Fig. 1:**
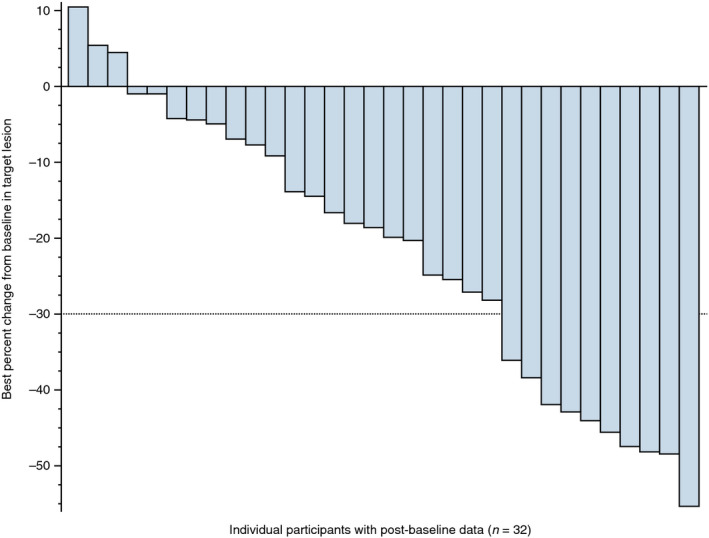
Waterfall plot of the best percentage change in the target lesion size (full analysis set). Change in the target lesion calculated by the sum of product diameters. Two patients with no target lesions and one participant with no post‐baseline tumor assessment results were excluded.

ORs were confirmed in seven patients; all of which were PRs (Table 2). The ORR was 20.0% (90% CI 9.8–34.3%). The lower limit of the 90% CI (9.8%) exceeded the prespecified threshold of 3%. ORs were confirmed in most subgroups regardless of demographics and baseline characteristics. The point estimates for ORR ranged 11.1–36.4% in subgroups with ≥10 patients (Table S1). A total of 30 patients had the best overall response of CR, PR or SD, resulting in a CBR of 85.7% (95% CI 69.7–95.2%). Of the 32 evaluable patients, 29 had a post‐baseline reduction in the sum of lesion diameters (Fig. 1). At data cut‐off, 24 (68.6%) patients were continuing to receive the study drug, and seven (20.0%) were being followed up in the post‐treatment period (Fig. S1).

**Table 2:** Tumor response by IRC in Japanese patients receiving at least one dose of cabozantinib (full analysis set)

Response	*n* = 35
Best overall response, *n* (%)	
Confirmed CR	0
Confirmed PR	7 (20.0)
SD	23 (65.7)
Progressive disease	4 (11.4)
Not evaluable	0
Missing	1 (2.9)
ORR	
*n* (%)	7 (20.0)
90% CI	(9.8–34.3)
CBR	
*n* (%)	30 (85.7)
95% CI	(69.7–95.2)

**Fig. 2:**
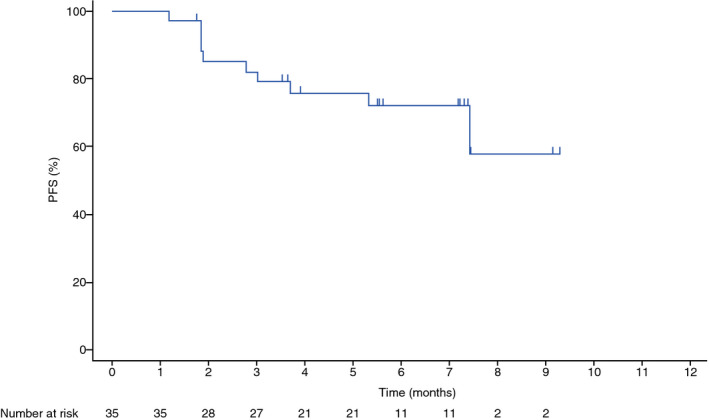
Kaplan–Meier plot of PFS per RECIST 1.1 (full analysis set).

**Fig. 3:**
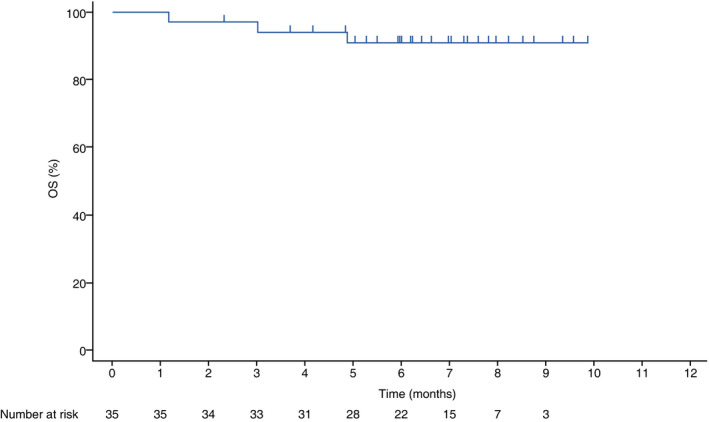
Kaplan–Meier plot of OS (full analysis set).

A total of 10 (28.6%) PFS events were recorded, including two deaths and eight occurrences of disease progression. Three (8.6%) deaths occurred by 23 October 2018 (data cutoff) in the OS analysis. The follow‐up time from enrollment of the last participant through data cut‐off was approximately 4 months. The median PFS was not reached (Fig. 2). The 6‐month PFS estimate was 72.3% (95% CI 53.3–84.6%). At data cut‐off, the median OS was also not reached (Fig. 3).

### PK assessment

Mean plasma concentrations of cabozantinib in all patients were in line with dose adjustments throughout the study. The mean (standard deviation) cabozantinib plasma concentrations in the full analysis set at weeks 3, 5 and 9 were 2078 (±899.71) ng/mL (*n* = 35), 1617 (±803.94) ng/mL (*n* = 34) and 1041 (±689.37) ng/mL (*n* = 34), respectively. For patients who had received cabozantinib 60 mg once daily for 14 or 15 days consecutively until each PK sampling point, the mean plasma concentrations of cabozantinib at steady‐state (assuming this had been reached) were 2317, 1733 and 1559 ng/mL at week 3, 5 and 9, respectively (Table 3).

**Table 3:** PK assessment (full analysis set)

	Week 3 (*n* = 35)	Week 5 (*n* = 34)	Week 9 (*n* = 34)
All patients			
Mean dose, mg (SD)	54.29 (17.20)	40.59 (24.86)	25.88 (23.37)
Mean plasma concentration, ng/mL (SD)	2078 (899.71)	1617 (803.94)	1041 (689.37)
Patients who achieved steady‐state at 60 mg†			
Mean plasma concentration, ng/mL (SD)	2317 (766.32)	1733 (662.77)	1559 (524.31)

† Patients who had cabozantinib 60 mg once daily for 14 or 15 days consecutively until each PK sampling point; the number at week 1 was 25, 19 at week 3 and 7 at week 5.

### Safety assessment

All patients experienced one or more AEs, and 68.6% reported grade ≥3 AEs (24/35; Table 4). Three deaths were reported that were attributed to progressive disease; one death occurred within 30 days and two deaths beyond 30 days, after the last dose of study drug. Grade ≥3 AEs were reported for 60% of patients with prior exposure to immuno‐oncology therapies in a post‐hoc analysis.

**Table 4:** Summary of frequent AEs (>10% of patients) in Japanese patients receiving at least one dose of cabozantinib (safety analysis set)

Preferred term, *n* (%)	Overall *n* = 35	
	Grade	
	All	Grade ≥3
Patients with any TEAEs	35 (100.0)	24 (68.6)
TEAEs in ≥10% of participants		
Palmar‐plantar erythrodysesthesia syndrome	22 (62.9)	3 (8.6)
Diarrhea	21 (60.0)	3 (8.6)
Hypertension	14 (40.0)	4 (11.4)
Proteinuria	14 (40.0)	3 (8.6)
Stomatitis	14 (40.0)	0
Dysgeusia	12 (34.3)	0
Hepatic function abnormal	12 (34.3)	3 (8.6)
Aspartate aminotransferase increased	9 (25.7)	1 (2.9)
Decreased appetite	9 (25.7)	3 (8.6)
Malaise	9 (25.7)	0
Weight decreased	9 (25.7)	1 (2.9)
Alanine aminotransferase increased	7 (20.0)	1 (2.9)
Constipation	7 (20.0)	0
Pyrexia	7 (20.0)	1 (2.9)
Dysphonia	6 (17.1)	0
Fatigue	6 (17.1)	3 (8.6)
Nausea	6 (17.1)	0
Vomiting	6 (17.1)	0
Amylase increased	5 (14.3)	1 (2.9)
Cancer pain	5 (14.3)	0
Hair color changes	5 (14.3)	0
Hypothyroidism	5 (14.3)	0
Insomnia	5 (14.3)	0
Rash	5 (14.3)	0
Blood thyroid stimulating hormone increased	4 (11.4)	0
Nasopharyngitis	4 (11.4)	0
Serum chemistry and hematology parameters in ≥10% of participants		
Lactate dehydrogenase increased	35 (100)	3 (8.6)
Creatinine increased	33 (94.3)	0
Urine protein‐to‐creatinine ratio increased	32 (91.4)	2 (5.7)
Hemoglobin decreased	29 (82.9)	1 (2.9)
Aspartate aminotransferase increased	27 (77.1)	3 (8.6)
Magnesium decreased	26 (74.3)	1 (2.9)
Albumin decreased	25 (71.4)	1 (2.9)
Alanine aminotransferase increased	24 (68.6)	3 (8.6)
Alkaline phosphatase increased	20 (57.1)	1 (2.9)
Amylase increased	18 (51.4)	2 (5.7)
Glucose increased	18 (51.4)	1 (2.9)
Phosphate decreased	17 (48.6)	5 (14.3)
Gamma glutamyl transferase increased	16 (45.7)	0
Sodium decreased	15 (42.9)	0
Lymphocytes decreased	12 (34.3)	5 (14.3)
Lipase increased	9 (25.7)	4 (11.4)
Platelets decreased	7 (20.0)	1 (2.9)
White blood cells decreased	6 (17.1)	0
Total bilirubin increased	5 (14.3)	0
Potassium increased	4 (11.4)	2 (5.7)
Magnesium increased	4 (11.4)	1 (2.9)
Potassium decreased	4 (11.4)	0

Dose modifications were permitted; 32 patients (91.4%) required dose modification due to an AE, with a median time to first dose modification of 25.0 days (range 2–87 days). Two patients (5.7%) discontinued due to AEs, including one each for gastric fistula and proteinuria.

## Discussion

In this phase II, open‐label, single‐arm study of cabozantinib of Japanese patients with advanced RCC and prior systemic therapy with VEGFR‐TKIs, ORR was 20.0% (90% CI 9.8–34.3%). This result is comparable to that observed in studies of cabozantinib in non‐Japanese patients with advanced RCC.^11^, ^12^ The safety and tolerability profile of cabozantinib in the present study was also consistent with previous reports.^11^, ^12^ Although all patients experienced TEAEs, the majority were managed through supportive treatment and/or dose modification.

The present study was designed to bridge the results from METEOR to the Japanese RCC population.^12^ The patient populations were similar, although the current population had a higher overall risk (68% had the Memorial Sloan Kettering Cancer Center intermediate or poor risk *vs* 54% for METEOR).^12^ Prior therapy use was similar in both studies, with approximately 70% receiving one or more TKIs and approximately 30% two or more TKIs.^15^ Nevertheless, the efficacy results in the present study are encouraging and generally consistent with the activity shown in non‐Japanese patients.^11^, ^12^, ^13^ All ORs were PR, and the proportion of patients with progressive disease as the best overall response (11.4%) was similar to METEOR (12%).^12^ Of interest, three patients experienced no anti‐tumor effect. There was no apparent correlation between response and baseline characteristics; future biomarker analysis is warranted.

METEOR reported better response rates with cabozantinib than with everolimus across most subgroups, including the Memorial Sloan Kettering Cancer Center risk classification. Even in patients with bone and visceral metastases known to contribute to poor prognosis, the PFS HRs were 0.33 (95% CI 0.21–0.51) and 0.48 (95% CI 0.38–0.60), respectively.^12^ In the present analysis, ORs were observed in most subgroups; however, small patient numbers limited the interpretation of the results. The ORR in subgroups with ≥10 patients ranged 11.1–36.4%, indicating efficacy regardless of risk classification, and number or class of prior treatments. No ORs were observed in patients with bone metastases (*n* = 8), but benefit was shown in those with lung metastases, and objective response was observed in two out of nine patients with liver metastases. Results from subgroup analyses were generally consistent with the overall cohort.

The most frequent AEs observed in this study included palmar‐plantar erythrodysesthesia syndrome (62.9%), diarrhea (60%), hypertension (40%), proteinuria (40%) and stomatitis (40%) in any grade. These were 42%, 74%, 37%, 12% and 22% in METEOR, respectively. Grade ≥3 AEs in the two studies were comparable.^12^ No between‐study differences were observed in abnormal laboratory values. The safety and tolerability of cabozantinib in this study were consistent with previously reported cabozantinib data and comparable with other TKIs in Japanese populations.^16^, ^17^

The median daily dose of cabozantinib in the present study (26 mg; median dose intensity 43%) was lower than that in METEOR (43 mg, relative dose intensity 71%),^12^ potentially due to discrepancies in the PK profile of different patient groups; steady‐state plasma exposures of cabozantinib in Japanese patients have been shown to be approximately 30% higher than those in their non‐Japanese counterparts.^14^ As the risk of AEs is dependent on cabozantinib exposure,^18^ more patients in the present study required dose adjustments than in METEOR (91% *vs* 62%).^12^ Nevertheless, few cabozantinib‐treated patients discontinued therapy due to AEs in this study, suggesting that many AEs can be effectively managed by dose adjustment. These data are also consistent with those of other TKIs in Japanese patients.^19^

The present study reported an ORR of 20% regardless of prior administration of immuno‐oncology therapies. Additionally, ad hoc analyses showed that three out of 10 cases in which the most recent treatment included an immuno‐oncology therapy resulted in an objective response. The overall AE profile of patients with prior exposure to immuno‐oncology therapies was similar to the main study population. Although definitive conclusions cannot be drawn due to the small patient number, these data suggest that cabozantinib might be a viable option after treatment involving VEGFR‐TKIs and/or immuno‐oncology agents. In fact, in the recently updated European Society for Medical Oncology and the National Comprehensive Cancer Network recommendations, cabozantinib was recommended as a second‐line treatment of clear cell RCC after treatment with TKI or immune‐oncology therapy.^20^, ^21^

A major strength of the present study is the alignment of study design to METEOR, allowing extrapolation of findings to Japanese patients despite the small sample size, the use of an open‐label, single‐arm design and a short duration of follow up, which might have impacted the ability to calculate PFS and OS. Although there are data from Japan suggesting that the AEs experienced with prior lines of TKI should not limit future TKI selection, more data are required to understand the profile and consequences for cabozantinib in Japanese patients.^22^

In summary, the present study showed that cabozantinib provided clinical benefits in Japanese patients with RCC, with similar efficacy and safety to the non‐Japanese population. TEAEs that occurred more frequently in Japanese than in non‐Japanese patients were effectively managed with dose adjustment and supportive care.

## Conflict of interest

Yoshihiko Tomita received honoraria from Astellas Medivation, Bristol‐Myers Squibb, Novartis, Ono Pharmaceutical and Pfizer; consulting fees from Novartis, Ono Pharmaceutical and Taiho Pharmaceutical; and grants or funds from Takeda Pharmaceutical, Astellas Medivation, AstraZeneca, Ono Pharmaceutical, Pfizer and Chugai Pharmaceutical. Katsunori Tatsugami received lecture fees from Ono Pharmaceutical and Bristol‐Myers Squibb. Noboru Nakaigawa, Takahiro Osawa, Naoto Sassa and Yasuhide Miyoshi have no potential conflicts to report. Mototsugu Oya received honoraria from Pfizer, Novartis, Bayer, Ono Pharmaceutical and Bristol‐Myers Squibb; consulting fees from Bayer; and grants or funds from Pfizer and Novartis. Hiroomi Kanayama received consulting fees from Takeda Pharmaceutical; grants or funds from Takeda Pharmaceutical, Astellas Pharmaceutical, Ono Pharmaceutical, Taiho Pharmaceutical, Novartis, Pfizer, MSD, Sanofi and Nihon Medi‐Physics; and lecture fees from Takeda Pharmaceutical, Astellas Pharmaceutical, Pfizer, Bayer, Ono Pharmaceutical, Janssen Pharmaceutical and MSD. Chihiro Nakayama Kondoh received honoraria from Takeda Pharmaceutical, Chugai Pharmaceutical, Bristol‐Myers Squibb, MSD and Eisai Company. Kazuo Nishimura received honoraria from Astellas Pharmaceutical and Novartis; consulting fees from Astellas Pharmaceutical, AstraZeneca and Janssen Pharmaceutical; and grants or funds from Bayer. Masahiro Nozawa received lecture fees from Bristol‐Myers Squibb, Ono Pharmaceutical and Novartis. Naoya Masumori received honoraria from Takeda Pharmaceutical, and grants or funds from Ono Pharmaceutical and Takeda Pharmaceutical. Shingo Kuroda and Akiko Kimura are employees of Takeda Pharmaceutical. Shingo Tanaka worked with Takeda Pharmaceutical as a contracted employee of a CRO to support their drug development. Satoshi Tamada received fees for lectures, and advisory boards from Bayer, Bristol‐Myers Squibb, MSD, Novartis, Ono Pharmaceutical, Pfizer and Takeda Pharmaceutical.

## Supporting information


**Figure S1**. Best response to cabozantinib therapy and duration of treatment.

**Table S1**. ORR per RECIST 1.1 in Japanese patients receiving cabozantinib by subgroup in the full analysis set (as adjudicated by IRC).
