# Supplementary figures and images for: Cabozantinib in advanced renal cell carcinoma: A phase II, open‐label, single‐arm study of Japanese patients
Source: Int J Urol. 2020 Aug 12;27(11):952–9. doi: 10.1111/iju.14329 (PMC7689847; doi:10.1111/iju.14329)

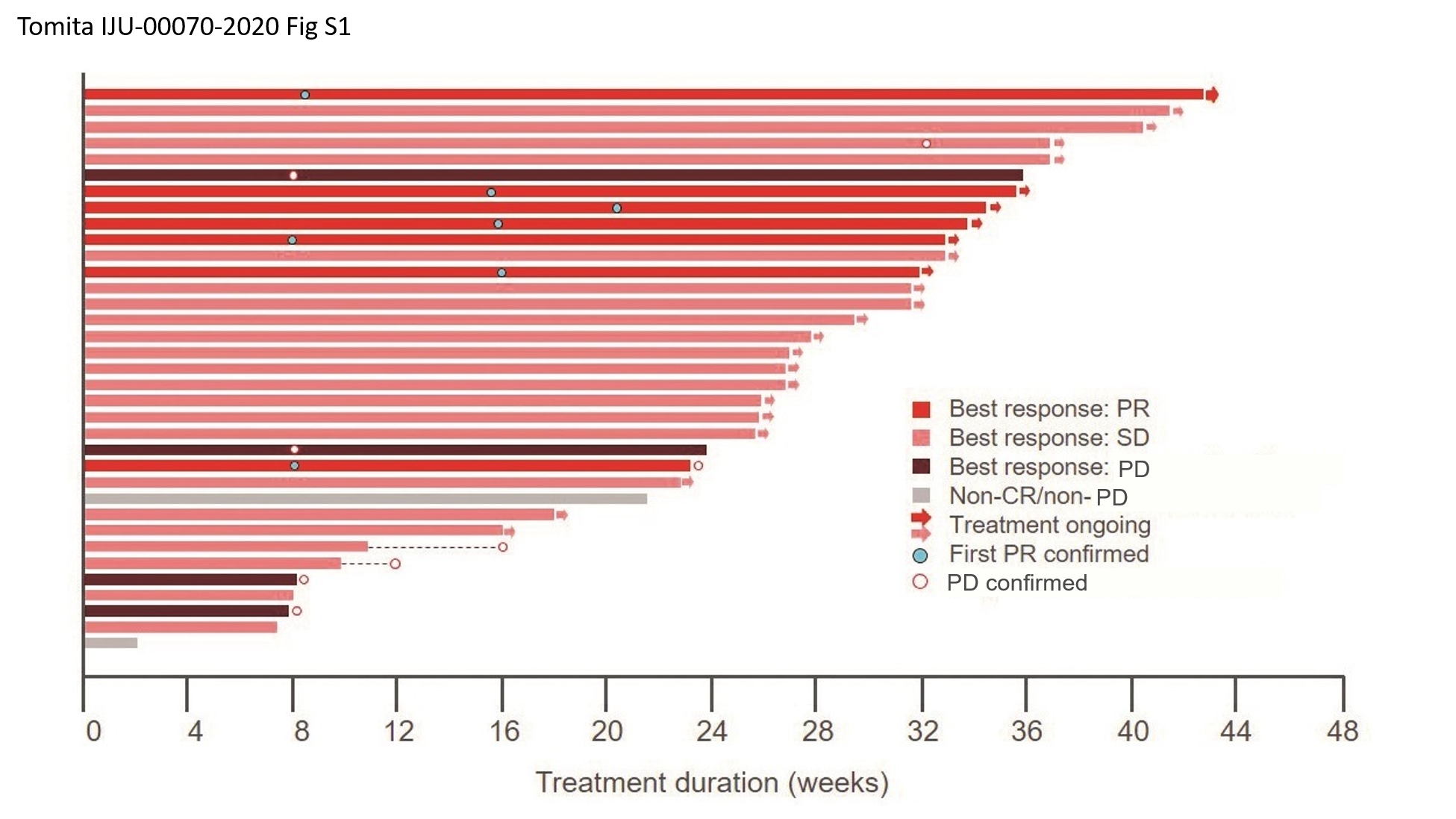

Supplement: Supplementary file 1 — Figure S1. Best response to cabozantinib therapy and duration of treatment. [file IJU-27-952-s001.png]
