# Supplementary material for: Cabozantinib in advanced renal cell carcinoma: A phase II, open‐label, single‐arm study of Japanese patients
Source: Int J Urol. 2020 Aug 12;27(11):952–9. doi: 10.1111/iju.14329 (PMC7689847; doi:10.1111/iju.14329)
Supplement: Supplementary file 2 — Table S1. ORR per RECIST 1.1 in Japanese patients receiving cabozantinib by subgroup in the full analysis set (as adjudicated by IRC). [file IJU-27-952-s002.docx]

**Table S1** ORR per RECIST 1.1 in Japanese patients receiving cabozantinib by subgroup in the full analysis set (as adjudicated by IRC)

| Subgroup | *N* | ORR, *n* (%) | 90% CI |
| --- | --- | --- | --- |
| Overall | 35 | 7 (20.0) | (9.8–34.3) |
| Age (years)  <65  ≥65 | 19  16 | 4 (21.1)  3 (18.8) | (7.5–41.9)  (5.3–41. 7) |
| Gender  Male  Female | 24  11 | 5 (20.8)  2 (18.2) | (8.6–38.9)  (3.3–47.0) |
| Time from diagnosis to enrollment  <1 year  ≥1 year | 4  31 | 1 (25.0)  6 (19.4) | (1.3–75.1)  (8.8–34.7) |
| Time from radiographic progression after most recent VEGFR-TKI to enrollment  <3 months  ≥3 months | 25  10 | 4 (16.0)  3 (30.0) | (5.7–33.0)  (8.7–60.7) |
| Number of involved organs  1  2  ≥3 | 6  11  18 | 1 (16.7)  2 (18.2)  4 (22.2) | (0.9–58.2)  (3.3–47.0)  (8.0–43.9) |
| MSKCC risk factor  Favourable  Intermediate  Poor | 11  19  5 | 2 (18.2)  4 (21.1)  1 (20.0) | (3.3–47.0)  (7.5–41.9)  (1.0–65.7) |
| IMDC criteria  Favorable  Intermediate  Poor | 6  22  7 | 0  6 (27.3)  1 (14.3) | (0.0–39.3)  (12.6–46.8)  (0.7–52.1) |
| ECOG PS  0  1  ≥2 | 26  9  0 | 6 (23.1)  1 (11.1)  0 | (10.6–40.5)  (0.6–42.9)  - |
| Prior nephrectomy  No  Yes | 1  34 | 0  7 (20.6) | (0.0–95.0)  (10.1–35.2) |
| Number of prior VEGFR-TKI agents  1  ≥2 | 24  11 | 4 (16.7)  3 (27.3) | (5.9–34.2)  (7.9–56.4) |
| Prior treatment with anti-PD-1, anti-PD-L1/L2 agents  No  Yes | 20  15 | 4 (20.0)  3 (20.0) | (7.1–40.1)  (5.7–44.0) |
